# Preclinical and clinical studies of CAR-NK-cell therapies for malignancies

**DOI:** 10.3389/fimmu.2022.992232

**Published:** 2022-10-24

**Authors:** Hongwen Li, Wenting Song, Zhaoming Li, Mingzhi Zhang

**Affiliations:** ^1^ Department of Oncology, The First Affiliated Hospital of Zhengzhou University, Zhengzhou, Henan, China; ^2^ State Key Laboratory of Esophageal Cancer Prevention and Treatment and Henan Key Laboratory for Esophageal Cancer Research, The First Affiliated Hospital of Zhengzhou University, Zhengzhou, Henan, China; ^3^ Translational Medical Center, The First Affiliated Hospital of Zhengzhou University, Zhengzhou, Henan, China; ^4^ Academy of Medical Sciences of Zhengzhou University, Zhengzhou, Henan, China

**Keywords:** chimeric antigen receptor, T cells, natural killer cells, immunotherapy, malignancies

## Abstract

The development of chimeric antigen receptor T (CAR-T) cell therapy, a specific type of immunotherapy, in recent decades was a fantastic breakthrough for the treatment of hematological malignancies. However, difficulties in collecting normal T cells from patients and the time cost of manufacturing CAR-T cells have limited the application of CAR-T-cell therapy. In addition, the termination of related clinical trials on universal CAR-T cell therapy has made further research more difficult. Natural killer (NK) cells have drawn great attention in recent years. Chimeric antigen receptor-NK (CAR-NK) cell therapy is a promising strategy in the treatment of malignant tumors because of its lack of potential for causing graft-versus-host disease (GVHD). In this review, we will address the advances in and achievements of CAR-NK cell therapy.

## Introduction

In recent decades, CAR-T-cell therapy was a research focus and was thought to be a promising targeted immunotherapy, especially in the treatment of relapsed and refractory B-cell malignant tumors. To date, two CD19-CAR-T-cell therapies have been approved for the treatment of acute lymphocytic leukemia (ALL) and diffuse large B-cell lymphoma (DLBCL) (1). Studies of CAR-T cells targeting CD38 and BCMA for the treatment of multiple myeloma (MM) have been implemented in clinical trials (2). However, CAR-T cell therapy is still facing several problems. The FDA has terminated all clinical trials concerning universal CAR-T-cell therapy due to safety consideration and related increased attention on gene editing. It is also difficult to collect sufficient numbers of T lymphocytes from patients who have been heavily pretreated. Furthermore, several weeks of CAR-T-cell preparation time hinder the use of this therapy to patients with rapid disease progression (3). In addition, cytokine release syndrome (CRS) and neurological toxicity (NT), the most common adverse events of CAR-T-cell therapy, are life-threatening (4). All of these factors may restrict further clinical applications of CAR-T-cell therapy.

In recent years, NK cells have been regarded as an alternative to T cells due to their accessibility and safety (5). Considering the short duration *in vivo*, the cytotoxicity and adverse events of CAR-NK-cell therapy are better manageable than those of CAR-T cell therapy. Moreover, the lower incidence of GVHD induced by NK cells makes them a promising immunotherapy for allogenic cell transplantation (6). CAR-NK-cell therapy has thus become a research hotspot and new strategy for malignancies.

In this review, we will discuss the similarities and differences between CAR-T cells and CAR-NK cells and focus on recent advances and preclinical studies of CAR-NK cells.

## The biological characteristics of NK cells

NK cells are innate immune effectors and are found mainly in the bone marrow, peripheral blood, spleen and liver (7). NK cells possess cytotoxic features similar to those of CD8+ T cells and play important roles in tumor immunology. CD8+ T-cell-mediated cytotoxicity relies on the combination of the T-cell receptor (TCR) and an antigen presented by major histocompatibility complex-I (MHC-I). NK cells can recognize MHC-I expressed on healthy cells and avoid attacking them (8, 9). Tumor cells can down-modulate MHC-I to escape CD8+ T-cell-mediated cytotoxicity, while NK cells can be activated through the loss of MHC-I and control the proliferation and metastasis of tumors (8, 10). Thus, NK cells have more specific anti-tumor effects and are associated with fewer off-target complications (9, 11).

The activation of NK cells can be mediated through different pathways, including signals from Toll-like receptors (TLRs) recognizing pathogen-associated molecular patterns (PAMPs), cytokines such as interleukin (IL)-2 or IL-15, and interplay between activating and inhibitory receptors (7, 12, 13). Activating NK-cell receptors include members of the natural cytotoxicity receptor (NCR) family (NKp30, NKp44 and NKp46), C-type lectin-like activating receptors (NKG2C and NKG2D), activating killer immunoglobulin receptors (KIR2DS1, KIR2DS4 and KIR2DL4) and costimulatory receptor DNAX accessory molecule 1 (DNAM-1) (14). While killer cell immunoglobulin-like receptors (KIRs) and the heterodimeric C-type lectin receptor NKG2A are inhibitory receptors associated with the tolerance of NK cells to normal cells (14).

## The sources of NK cells for immunotherapy

NK cells for preclinical studies and clinical therapy may be derived from a wide range of sources, such as peripheral blood (PB), cord blood (CB), hematopoietic stem cells (HSCs), induced pluripotent stem cells (iPSCs) and NK-cell lines (15–19).

The most accessible source of NK cells is peripheral blood. However, a number of issues limit the use of NK cells from peripheral blood, including the high monetary and time costs, low cell proliferation capacity and short survival time (20). The expression of genes related to the cell cycle and cell proliferation is higher in NK cells from umbilical cord blood (UCB) than in those from peripheral blood (21). Furthermore, the advantages of UCB-derived NK cells, including the convenience of collection and low associated incidence of GVHD, make UCB a better source of NK cells than PB (22, 23). In addition, human stem and progenitor cells (HSPCs) isolated from cord blood can also be derived into NK cells with the stimulation of various growth factors and cytokines, including IL-2, IL-7 and IL-15 (24). Similarly, NK cells can also be derived from iPSCs in the presence of these stimulators (25).

NK-cell lines, mostly derived from NK/T-cell lymphoma (NKTCL) patients, such as the NK-92 and KHYG-1 cell lines, may be a potential rapid and abundant source for NK cells for immunotherapy (26, 27). These cell lines are easily transduced and maintain cytotoxicity during expansion. The NK-92 cell line, obtained from a good manufacturing practice (GMP)-compliant master cell bank and treated in a GMP-compliant procedure, is the only cell line approved by the FDA for clinical use (28, 29). Since the first report of the transfusion of irradiated NK-92 cells for adoptive immunotherapy of malignancies (30) and the first CAR-NK-92 cells targeting HER-2 (31), NK-92 cells has been applied in several clinical trials, and some encouraging results have been achieved in the treatment of refractory lymphoma, multiple myeloma and other solid tumors. Several patients even achieved a complete response (CR) (32–34). NK-cell lines must be irradiated before infusion due to the risk of tumor engraftment and tumorigenicity. The short lifespan of irradiated cells may result in treatment failure or a short duration of disease remission, thus limiting their clinical application (32, 33, 35).

## The similarities and differences between CAR-T cells and CAR-NK cells

**Figure 1:**
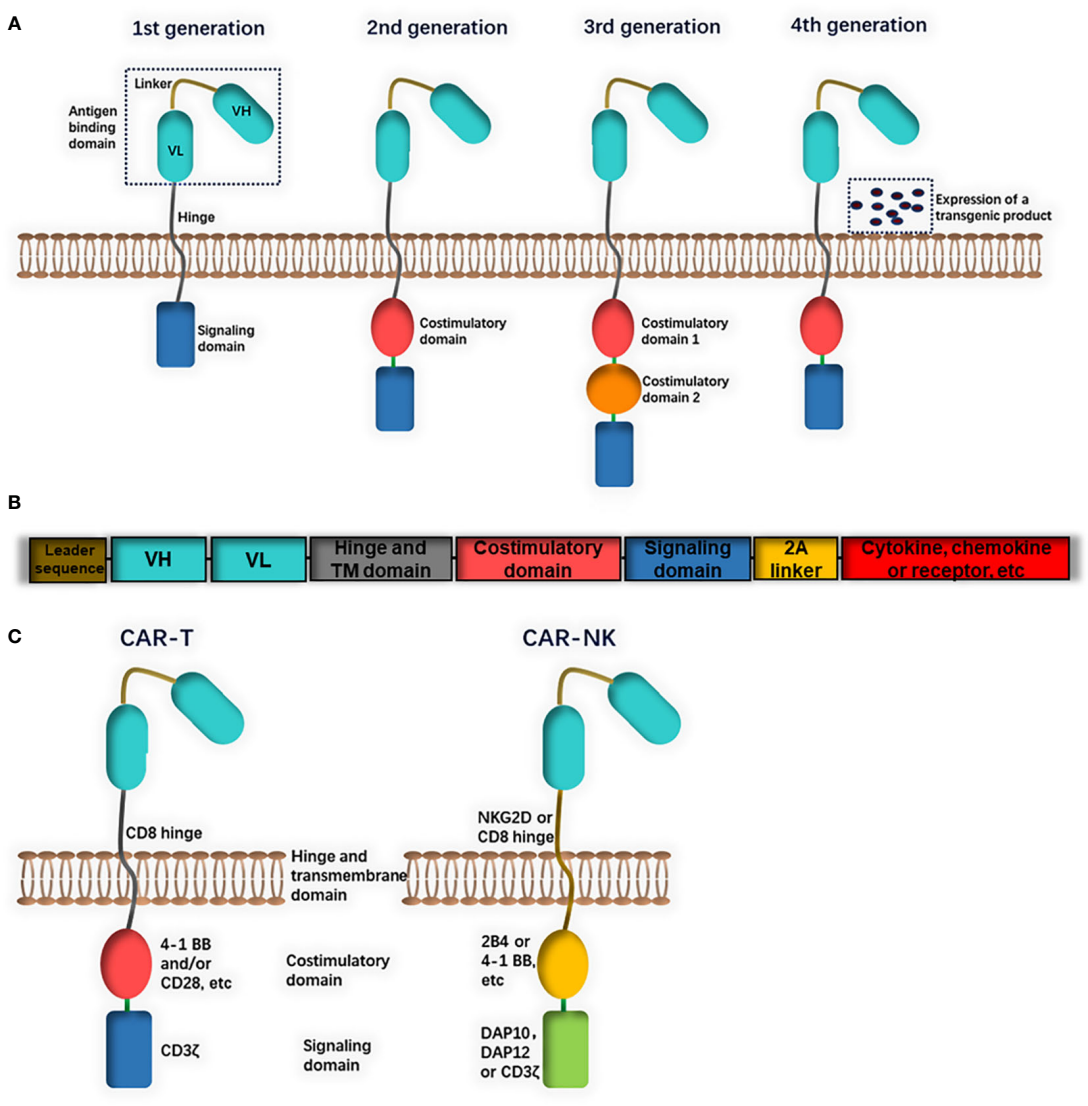
The structure of chimeric antigen receptors (CAR). **(A)** CAR consist of an extracellular antigen binding domain, a transmembrane hinge and intracellular domain. The extracellular domain could be a single chain fragment of variable region (ScFv) antibody or a functional domain of specific ligand. The intracellular domain is composed of a signaling domain (first generation) and one costimulatory domain (second generation) or two (third generation). **(B)** Fourth generation CARs include a constitutive or inducible expression of a transgenic product (cytokine, chemokine or receptor, etc.). **(C)** Differences in CAR constructs between CAR-T and CAR-NK: CAR-T cells usually contains a CD8 transmembrane domain, CD3ζ signaling domain and 4-1BB and/or CD28 costimulatory domain. CAR-NK cells may be with different domains (for example, NKG2D transmembrane domain, DAP10 or DAP12 signaling domain and 2B4 costimulatory domain).

CARs consist of an extracellular domain (a single-chain variable antibody fragment (scFv) or a functional domain of a specific ligand) for the identification of target antigens, a transmembrane region and an intracellular domain (36). The intracellular domain of CAR-T cells is composed of CD3ζ activation signaling (first generation of CARs) and costimulatory molecules (CD28, 4-1BB or CD134) (second or third generation of CARs) (**Figure 1A**). Based on NK-cell characteristics, several CAR-NK cells contain DNAX-activation protein (DAP) 10 or DAP12 as an intracellular domain (**Figure 1C**). DAP12 and NKG2D are expressed on NK cells and participate in the activation of downstream signals, while DAP10 is necessary for NKG2D costimulatory signaling. These CAR-NK cells were mainly designed for the treatment of both leukemia and solid tumors and showed strong anti-tumor effects (37, 38). A lack of cytokines such as IL-2 or IL-15 may lead to the short *in vivo* lifetime of NK cells. NK cells can be engineered to both express CARs and autonomously produce IL-2 or IL-15 (fourth generation of CARs), thus enhancing their persistence and proliferation (**Figure 1B**) (39, 40).

Lentivirus-based vectors have been extensively used in CAR gene transduction of T cells. Compared with T cells, NK cells showed resistance to viral transfection and lower transduction efficiency, which may be due to the natural capacity of NK cells to defend against viral infection (41, 42). Other approaches, including retroviral vectors, transposon vectors and the electroporation of DNA or mRNA plasmids, are alternative ways to transfer the CAR gene into NK cells (43–48).

CAR-T cells can kill tumor cells with specific target antigens through active cell lysis and the production of cytokines, including IL-1α, IL-2, IL-6, IL-8, IL-10, and tumor necrosis factor-α (TNF-α) (6, 49). However, these cytokines are also highly associated with CRS and severe neurotoxicity (49). CAR-NK cells secrete a different cytokine profile, such as IFN-γ and GM-CSF, which are associated with a lower risk of CRS and neurotoxicity (50). In addition, CAR-NK cells can lyse tumor cells directly by releasing cytoplasmic granules containing perforin and granzyme or inducing tumor cell apoptosis by expression of Fas ligand or TNF-related apoptosis-inducing ligand (TRAIL) (51). NK cells also participate in antibody-dependent cellular cytotoxicity (ADCC) (52). NK cells can activate and interact with other immune cells, such as T cells, dendritic cells and macrophages (53). All these features enable them to exert anti-tumor activity in pathways other than the CAR-specific pathway and reduce the risk of relapse or resistance mediated by target antigen escape (54–56).

## Preclinical studies of CAR-NK cells in the treatment of hematopoietic malignancies

NK cells have been engineered to express CARs to redirect their activity against B-cell malignancies. To date, CD19 is the most common target in both preclinical and clinical studies of CAR-T-cell therapy. Similarly, a number of preclinical studies of CAR-NK therapy have focused on this target. NK-92 cells engineered with CARs recognizing CD19 showed increased cytotoxicity against B-cell malignancies (57, 58). CD19-CAR-NK cells from other cell sources, including PB, iPSCs and CB, also showed activity against B-cell malignancies *in vitro* (40, 59, 60). Other molecules, including CD20 and Flt3, were also developed as specific targets for CAR-NK immunotherapy against B-cell tumors (61, 62).

CD38 and CD138 are classic markers of plasma cells and are highly expressed in multiple myeloma (MM). Although CD38-CAR-T-cell therapy for MM and CD38-CAR-NK-cell therapy for acute myeloid leukemia (AML) have been reported in several studies (63, 64), CD38-CAR-NK cells have not been evaluated for the treatment of multiple myeloma. Jiang et al. developed CD138-targeting CAR-NK cells and demonstrated enhanced anti-tumor activity *in vitro* and in xenograft mouse models (65). B-cell maturation antigen (BCMA) is another ideal target for CAR cell therapy due to its restricted expression in B-cell lineage cells. BCMA-CAR-NK cells modified with CXCR4 significantly reduced the tumor burden and extended the survival of tumor-bearing mice (66). Signaling lymphocytic activation molecule family member 7 (SLAMF7 or CS1) is another potential target for its high expression in plasma cells and MM. Second-generation CS1-specific CAR-NK-92 cells were established by Chu et al. and showed cytotoxicity against CS1-positive MM cells and xenograft models (67).

To date, T-cell malignancies, including peripheral T-cell lymphoma and T-cell acute lymphoblastic leukemia (T-ALL), remains a refractory disease. Three CAR-NK cell therapies targeting CD3, CD5 and CD7 have been investigated for the treatment of T-cell malignancies. These modified CAR-NK-92 cells showed significant anti-tumor cytotoxicity against T-cell lymphomas and T-ALL both *in vitro* and *in vivo* (68–70).

In addition to specific tumor markers, antigens that are widely expressed in multiple malignancies have been developed as immunotherapy targets. For example, NKG2D ligands are expressed on a variety of tumor cells. MHC class I chain-related protein A (MICA), an NKG2D ligand, has been identified on some leukemia cells and solid tumor cells, such as lung, breast, ovary and colon cancer cells (71–73). NKG2D ligands have also been detected on MM cells and glioma cells (74, 75). Leivas et al. developed engineered NK cells targeting NKG2D ligands in MM (76). Data from *in vitro* tests and mouse models showed enhanced anti-tumor activity of NKG2D-CAR-NK cells compared with memory CAR-T cells (76). Du et al. generated peripheral blood-derived NK cells coexpressing NKG2D-specific CAR and IL-15 and demonstrated their activity in lysing tumor cells both *in vitro* and in a xenograft AML model (77).

## Preclinical studies of CAR-NK cells in the treatment of solid tumors

Although CAR-T-cell therapies have achieved great progress in the treatment of hematological malignancies, their effect on solid malignancies has been poor. This poor efficacy may be due to the insufficient homing capacity and the immunosuppressive tumor microenvironment (78). Thus, CAR-NK cell therapies for solid tumors have become a promising immunotherapy strategy. Glioblastoma, breast cancer and ovarian cancer are the most widely researched solid tumors to determine the potential of CAR-NK-cell therapy (summarized in **Table 1**).

**Table 1:** Preclinical studies of CAR-NK cell therapy.

Malignancy	Target	Source of NK cells	Reference
**Hematological cancer**			
B-cell malignancies	CD19	NK-92, PB-NK or CB-NK	(40, 57–60)
	CD20	NK-92	(61)
	Flt3	NK-92	(62)
Multiple myeloma	CD138	NK-92	(65)
	BCMA	NK-92	(66)
	CS1	NK-92	(67)
	NKG2D	PB-NK	(77)
T-cell malignancies	CD3	NK-92	(68)
	CD5	NK-92	(69)
	CD7	NK-92	(70)
AML	NKG2D	PB-NK	(77)
**Solid cancer**			
Glioblastoma	HER2	NK-92	(79)
	EGFR and/or EGFRvIII	NK-92, NKL, KHYG1 or YTS	(80–84)
Breast cancer	HER2	NK-92	(29, 85, 86)
	EGFR and/or EGFRvIII	NK-92 or PB-NK	(87)
	EpCAM	NK-92	(88)
	TF	NK-92	(89)
	B7-H6	NK-92	(90)
Ovarian cancer	HLA-G	PB-NK	(91)
	CD24	NK-92	(92)
	CD44	NK-92	(93)
	CD133	NK-92	(94)
	Mesothelin	iPSC-NK or NK-92	(95, 96)
	αFR	NK-92	(97)

### Glioblastoma

Glioblastoma is the most common malignant primary cerebral tumor in adults. Even though patients undergo surgical resection and receive radio- and/or chemotherapy, the median survival time is approximately 15 months (98). Interleukin-13 receptor α2 (IL-13Rα2), epidermal growth factor receptor (EGFR), EGFR variant III (EGFRvIII) and growth factor receptor tyrosine kinase Erb2 (HER2) have been explored as immunotherapy targets for glioblastoma. They are overexpressed in 40-60% of glioblastoma patients, while these antigens are undetectable or only minimally expressed in normal brain tissue (99–102). IL-13Rα2 can enhance the invasiveness of glioblastoma (103). EGFRvIII drives tumorigenicity and mediates resistance to radiotherapy and chemotherapy (104, 105). Together, IL-13Rα2 and EGFRvIII can promote the proliferation of glioblastoma cells (103), while overexpression of HER2 contributes to malignant transformation (106).

There have been several preclinical studies of IL-13Rα2-specific CAR-T-cell therapy in the treatment of glioblastoma (107–110). Other studies demonstrated the significant cytotoxicity of CAR-T cells against EGFRvIII- or HER2-positive glioblastoma both *in vitro* and *in vivo* (111–114).

Until now, most preclinical studies of CAR-NK-cell therapy for glioblastoma were targeting EGFR, EGFRvIII and HER2. Different NK cells, including NK-92, NKL, KHYG-1 and YTS cells, engineered to target EGFR and/or EGFRvIII, showed enhanced cytotoxicity against glioblastoma both *in vitro* and *in vivo* (80–83). CAR-NK cells recognizing both EGFR and EGFRvIII showed stronger anti-tumor effects than single targeted NK cells (84). NK-92/5.28z cells, engineered HER2-specific NK cells with CD28 and CD3ζ signaling domains, have been demonstrated to have the ability to lyse HER2-positive glioblastoma cells *in vitro* and in orthotopic glioblastoma xenograft NSG mouse models (79).

### Breast cancer

As a very common malignancy in female patients, breast cancer is another solid tumor that is studied for CAR-NK-cell immunotherapy. Similar to glioblastoma, HER2, EGFR and EGFRvIII are also targets for breast cancer.

The anti-tumor activity of NK-92/5.28z cells was also evaluated in HER-2-positive breast cancer. Data revealed that tumor cells expressing HER-2 enhanced the proliferation and cytokine release (such as granzyme B, IFN-γ, IL-8 and IL-10) of NK-92/5.28z cells [87]. The modified NK-92 cells displayed significant cytotoxicity *in vitro* and in xenograft mouse models (85). NK-92 cells engineered to target HER2 developed by Liu et al. also demonstrated similar anti-tumor effects (86).

A second-generation CAR that can recognize both EGFR and EGFRvIII was constructed by Chen et al. (87). NK-92 cells transduced with this CAR showed enhanced cytotoxicity and production of IFN-γ against breast cancer cells. Xenograft mouse models of breast cancer brain metastasis were used for *in vivo* evaluation of anti-tumor activity. CAR-NK-92 cell infusion significantly suppressed tumor growth. Similarly, two EGFR-targeted CAR-NK cells were developed (87). Cytokine release and cytotoxicity assays were performed and revealed that EGFR-CAR NK cells specifically lysed triple-negative breast cancer cells *in vitro* and suppressed breast cancer cell line-derived xenograft and patient-derived xenograft (PDX) tumors in mouse models (87).

Epithelial cell adhesion molecule (EpCAM), tissue factor (TF) and B7-H6 have also been reported as targets for the treatment of breast cancer. Studies have shown the increased tumor killing ability of these CAR-NK-92 cells against breast cancer cells (88–90).

### Ovarian cancer

Ovarian cancer is a highly malignant tumor with a 5-year survival rate lower than 40% (115). Several studies have focused on CAR-NK immunotherapies for the treatment of ovarian cancer.

Human leukocyte antigen G (HLA-G) is a tumor-associated antigen (TAA) that is expressed on 40-100% of solid tumors and a limited subset of immune-privileged tissues and adult tissues, such as erythroid precursors and pancreatic islets (116, 117). Jan et al. developed CAR-NK cells targeting HLA-G and evaluated the synergy of CAR-NK cells combined with low-dose chemotherapy (118). Jan et al. developed CAR-NK cells targeting HLA-G and evaluated the synergy of CAR-NK cells combined with low-dose chemotherapy (116). Their study showed that pretreatment with low-dose chemotherapy can induce the overexpression of HLA-G, thus enhancing the anti-tumor cytotoxicity of HLA-G-CAR-NK cells (91).

Since cancer stem cells (CSC) play an important role in metastatic spread and chemoresistance in solid tumors, CSC markers such as CD24, CD44 and CD133 have been explored as specific targets for ovarian cancer immunotherapy (92–94). CAR-NK-92 cells targeting CD24, CD44 or CD133 have shown significant anti-tumor effects in preclinical studies (92–94).

Mesothelin and folate receptor alpha (αFR) are alternative targets that are overexpressed in ovarian cancer. Both iPSC-derived CAR-NK cells and NK-92 cell line-derived CAR-NK cells targeting mesothelin showed robust specific anti-tumor activity both *in vitro* and *in vivo* (95, 96). Ao et al. developed αFR-targeted CAR-NK-92 cells and demonstrated not only their antigen-specific cytotoxicity and proliferation *in vitro* but also their ability to eliminate cancer cells in mouse models (97).

## Clinical applications of CAR-NK cells

Since the first CAR-NK-cell clinical trials (NCT00995137, clinicaltrials.gov) started in 2009, there have been 39 studies registered in clinicaltrials.gov evaluating the feasibility, safety and efficacy of CAR-NK cells in the treatment of malignancies. Eight clinical trials sponsored by PersonGen BioTherapeutics and Asclepius Technology Company Group, including NCT02742727, NCT02839954, NCT02892695, NCT02944162, NCT03941457, NCT03931720, NCT03940820 and NCT03940833, which were estimated to be completed in 2018-2019, have been stopped updating for 3 years. It’s a pity that no data of these trials were reported till now. The rest of 31 trials were summarized in **Table 2**.

**Table 2:** Clinical trials for CAR-NK cell immunotherapy.

NO. NCT	Other Name/ID Numbers	States	Start Date	Phase	Disease	Target	Sponsor locations	NK source
NCT00995137	NKCD19 R01CA113482 NCI-2011-01226	Completed in May 2013.	October 2009	I	B-Lineage Acute Lymphoblastic Leukemia	CD19	St. Jude Children’s Research Hospital	PB-NK
NCT01974479	NKCARCD19	Suspended for an interim review of (CAR) CD19 research strategy	September 2013	I	B-Lineage Acute Lymphoblastic Leukemia	CD20	National University Health System, Singapore	PB-NK
NCT03056339	2016-0641 NCI-2018-01221	Active, not recruiting Primary results published.(119)	June 21, 2017	I and II	B Lymphoid Malignancies	CD19	M.D. Anderson Cancer Center	UCB-NK
NCT03383978	EudraCT 2016-000225-39	Recruiting	December 1, 2017	I	Glioblastoma	HER2	Johann Wolfgang Goethe University Hospital	NK-92
NCT03415100	NRC-NK-01	Completed Results submitted in February 2021	January 2, 2018	I	Metastatic Solid Tumors	NKG2D	The Third Affiliated Hospital of Guangzhou Medical University	PB-NK
NCT03656705	CNK-101	Enrolling by invitation	September 29, 2018	I	Non-small Cell Lung Carcinoma	PD-1	Xinxiang medical university	NK-92
NCT03692663	TABP EIC-01	Recruiting	December, 2018	Early I	Castration-resistant Prostate Cancer	PSMA	Allife Medical Science and Technology Co., Ltd.	Unknown
NCT03824964	CD19/CD22 CAR NK-BJZL-01	Unknown	February 1, 2019	Early I	Relapsed or Refractory B Cell Lymphoma	CD19/CD22	Allife Medical Science and Technology Co., Ltd.	Unknown
NCT03692767	CD22 CAR NK-BJZL-01	Unknown	March 2019	Early I	Relapsed and Refractory B Cell Lymphoma	CD22	Allife Medical Science and Technology Co., Ltd.	Unknown
NCT03690310	CD19 CAR NK-BJZL-01	Unknown	March 2019	Early I	Relapsed and Refractory B Cell Lymphoma	CD19	Allife Medical Science and Technology Co., Ltd.	Unknown
NCT03692637	Mesothelin Car NK-HNRM-01	Unknown	March 2019	Early I	Epithelial Ovarian Cancer	Mesothelin	Allife Medical Science and Technology Co., Ltd.	PB-NK
NCT04245722	FT596-101	Recruiting	March 19, 2020	I	B-Cell Lymphoma, Chronic Lymphocytic Leukemia	CD19	Fate Therapeutics	iPSC-NK
NCT04623944	NKX101-101	Recruiting	September 21, 2020	I	Adults With AML or MDS	NKG2D	Nkarta Inc.	PB-NK
NCT05215015	IBR733-T01 WX-IBR-7	Recruiting	November 30, 2020	Early I	Acute Myeloid Leukemia	CD33/CLL1	Wuxi People’s Hospital	Unknown
NCT04639739	CAR NK for NHL	Not yet recruiting	December 17, 2020	Early I	Relapsed or Refractory B Cell Non-Hodgkin Lymphoma	CD19	Xinqiao Hospital of Chongqing	Unknown
NCT04747093	ITNK-2021	Recruiting	January 29, 2021	I and II	B Cell Malignancies	CD19	Nanfang Hospital of Southern Medical University	Induced-T Cell Like NK cells
NCT04796675	CAR-NK-CD19 cells	Recruiting	April 10, 2021	I	B Lymphoid Malignancies	CD19	Wuhan Union Hospital, China	CB
NCT04887012	IR2021002168	Recruiting	May 1, 2021	I	Refractory or Relapsed B-cell Non Hodgkin Lymphoma	CD19	Second Affiliated Hospital, School of Medicine, Zhejiang University	PB-NK
NCT05020678	NKX019-101	Recruiting	August 20, 2021	I	Adults With B-cell Cancers	CD19	Nkarta Inc.	PB-NK
NCT05137275	IBR854-03	Recruiting	November 24, 2021	Early I	Locally Advanced or Metastatic Solid Tumors	5T4	Shanghai East Hospital	Unknown
NCT05008536	BCMA NK for MM	Recruiting	October 1, 2021	Early I	Relapsed or Refractory Multiple Myeloma	BCMA	Xinqiao Hospital of Chongqing	UCB-NK and CB-NK
NCT05247957	CARNK-001	Recruiting	October 13, 2021	I	Relapsed or Refractory Acute Myeloid Leukemia	NKG2D	Hangzhou Cheetah Cell Therapeutics Co., Ltd	UCB-NK
NCT05213195	CARNK-002	Recruiting	December 10, 2021	I	Refractory Metastatic Colorectal Cancer	NKG2D	Zhejiang University	Unknown
NCT04847466	10000096, 000096-C	Recruiting	December 14, 2021	II	Recurrent or Metastatic Gastric or Head and Neck Cancer	PD-L1	National Cancer Institute (NCI)	NK-92
NCT05008575	CD33 CAR NK-AML	Recruiting	December 23, 2021	I	Relapsed or Refractory Acute Myeloid Leukemia	CD33	Xinqiao Hospital of Chongqing	Unknown
NCT05194709	IBR854-T01, WX-IBR-8	Recruiting	December 30, 2021	Early I	Advanced Solid Tumors	5T4	Wuxi People’s Hospital	Unknown
NCT05379647	NK-002 (QN-019a)	Recruiting	November 4, 2021	I	B-Cell Malignancies	CD19	Zhejiang University	iPSC-NK
NCT05182073	FT576-101	Recruiting	November 24, 2021	I	Multiple Myeloma	BCMA	Fate Therapeutics	iPSC-NK
NCT05110742	2021-0526	Not yet recruiting	June 30, 2022	I and II	Relapse or Refractory Hematological Malignances	CD5	M.D. Anderson Cancer Center	CB-NK
NCT05092451	2021-0386	Not yet recruiting	August 1, 2022	I and II	Relapse or Refractory Hematological Malignances	CD70	M.D. Anderson Cancer Center	CB-NK
NCT05336409	CNTY-101-111-01	Not yet recruiting	December 2022	I	Relapsed or Refractory CD19-Positive B-Cell Malignancies	CD19	Century Therapeutics, Inc.	iPSC-NK

Allife Medical Science and Technology has just revised the completion date of NCT03692663. As for their other clinical trials, NCT03824964, NCT03692767, NCT03690310 and NCT03692637, we are looking forward to their renewal.

Similar to CAR-T-cell therapies, most CAR-NK-cell trials target markers on hematopoietic malignancies, such as CD19, CD20, CD22 and BCMA. Notably, there have been eight CAR-NK-cell clinical studies have focused on solid malignancies, which are thought to poorly responsive to CAR-T cells. These CAR-NK cells may target markers such as HER2, NKG2D, mesothelin and PSMA expressed on malignancies, including brain, prostate, ovarian, pancreatic and lung cancers (**Table 2**).

## Discussion

Studies in recent years suggest that CAR-NK-cell therapies may be equally effective as CAR-T-cell therapies. Compared with CAR-T cells, CAR-NK cells have multiple advantages for the treatment of malignancies. CAR-NK-cell therapy seldom causes severe CRS or neurotoxicity. The low associated risk of GVHD and the safety of allogeneic NK-cell infusion shorten the time of cell preparation, which greatly benefits patients with lymphopenia or rapid progression. However, several nonnegligible problems still exist. The best source of NK cells and their *in vitro* expansion strategy, and the most effective signaling domain for CAR activation still need to be elaborated. Antigen escape and tumor heterogeneity, the most common difficulties in immunotherapies, as well as *in vivo* duration, are also problems to be considered. CAR-NK-cell immunotherapy is still in its early stages. Strategies to improve the efficacy and safety of CAR-NK-cell immunotherapy must be further explored in the future.

## Author contributions

HL: conceptualization and writing original draft. WS: writing review and editing. ZL: writing review and editing. MZ: conceptualization, supervision, and writing – review and editing. All authors contributed to the article and approved the submitted version.

## Funding

The work was supported by the Oncology Department, State Key Laboratory of Esophageal Cancer Prevention & Treatment and Henan Key Laboratory for Esophageal Cancer Research, and the Medical Sciences Academy and Research Institute of Nephrology of Zhengzhou University. This work was supported by the National Natural Science Foundation of China (81970184; 82170183; U1904139; 82070209).

## Acknowledgments

I would like to show great gratitude to them all.

## Conflict of interest

The authors declare that the research was conducted in the absence of any commercial or financial relationships that could be construed as a potential conflict of interest.

## Publisher’s note

All claims expressed in this article are solely those of the authors and do not necessarily represent those of their affiliated organizations, or those of the publisher, the editors and the reviewers. Any product that may be evaluated in this article, or claim that may be made by its manufacturer, is not guaranteed or endorsed by the publisher.
